# Transcranial Direct-Current Stimulation as an Adjunct to Verb Network Strengthening Treatment in Post-stroke Chronic Aphasia: A Double-Blinded Randomized Feasibility Study

**DOI:** 10.3389/fneur.2022.722402

**Published:** 2022-03-02

**Authors:** Shereen J. Matar, Caroline Newton, Isaac O. Sorinola, Marousa Pavlou

**Affiliations:** ^1^Centre for Human & Applied Physiological Sciences, Faculty of Life Sciences & Medicine, King's College London, London, United Kingdom; ^2^Division of Psychology and Language Sciences, Faculty of Brain Sciences, University College London, London, United Kingdom; ^3^Department of Population Health Sciences, Faculty of Life Sciences & Medicine, King's College London, London, United Kingdom

**Keywords:** aphasia, stroke, language, transcranial direct current stimulation (tDCS), discourse, treatment, rehabilitation

## Abstract

**Background:**

Difficulties in discourse production are common in post-stroke chronic aphasia. Previous studies have found that speech and language therapy combined with transcranial direct-current stimulation (tDCS) may improve language skills like naming and enhance aphasia treatment outcomes. However, very few studies have investigated the effect of tDCS when combined with interventions for improving higher level language skills such as the Verb Network Strengthening Treatment (VNeST).

**Aims:**

This study aimed to determine the feasibility of anodal tDCS as an adjunct to VNeST to improve discourse production in post-stroke chronic aphasia.

**Methods:**

Six people with post-stroke chronic aphasia took part in this double-blinded randomized feasibility study. Participants were randomly allocated to either the experimental group receiving a 6-week block of once weekly VNeST sessions combined with active tDCS over the left inferior frontal gyrus (LIFG) or a control group that received VNeST with sham stimulation. Feasibility outcomes included screening, eligibility, retention, and completion rates, and adverse events. Preliminary response to intervention was also examined using discourse production, functional communication, quality of life, psychological state, and cognition outcomes.

**Results:**

Overall 19 individuals were screened and ten met the inclusion criteria. Six individuals provided consent and participated in the study giving a consent rate of 60%. Participant retention and completion rates were 100% and no adverse effects were reported. Exploratory analyses revealed promising changes (i.e., estimated large effect size) in discourse production measures across discourse language tasks and functional communication for the active tDCS group.

**Conclusions:**

Our results support the feasibility of tDCS as an adjunct to VNeST. Preliminary findings provide motivation for future large-scale studies to better understand the potential of tDCS as a safe and economical tool for enhancing rehabilitation in chronic aphasia.

## Introduction

Discourse is an integral part of everyday communication which allows humans to express their thoughts and feelings and effectively carry out daily activities (1, 2). Difficulties in discourse production (i.e., everyday use of language such as storytelling, giving instructions, conversing) are common in post-stroke aphasia and often impact psychosocial well-being and limit quality of life and social participation (3, 4). Speech and language therapy (SLT) has been recognized as the gold standard for treating aphasia including discourse impairments and intervention has been found to be effective (5, 6). However, intervention effect sizes are often small and naming remains the most regularly used outcome measure for aphasia recovery (3, 6–9). Treatments focused solely on word recovery can effectively target the essential components of communication (nouns, verbs etc.) but may fail to impact one's ability to produce sentences and longer utterances and therefore fail to address adequately the main objective of language intervention which is to improve everyday communication (7, 10, 11). Thus, there is a need for investigating new strategies which may enhance higher-level language skills important for every-day communication such as discourse production.

Non-invasive brain stimulation techniques like anodal tDCS which increases spontaneous cortical activity through subthreshold depolarization (12, 13) and promotes neuroplasticity (14, 15) may be effective in enhancing aphasia treatment effects in stroke survivors (16–19). People with post-stroke chronic aphasia have experienced improvement in naming accuracy and speed after application of anodal tDCS (16, 20, 21). Although, the majority of tDCS research has focused on word level recovery, a few studies have found that it may also improve language in the sentence and discourse context, particularly when applied over the left inferior frontal gyrus (LIFG). In a randomized cross-over study with neurotypical older adults the application of anodal tDCS on the LIFG resulted in significant improvements in recount, procedural and narrative discourse tasks compared to stimulation of the right IFG or sham (15). In post-stroke aphasia tDCS studies not working at the single word level have focused on conversation therapy. In a single case study, a participant with anomic type aphasia produced significantly more verbs in sentence production after application of anodal tDCS over the LIFG (Broca's area) when combined with sentence production training and conversational therapy (19), while in Marangolo et al. the same tDCS montage with conversational therapy in a group of eight and 12 participants, respectively resulted in greater language cohesion and increased informational content, verbs, and sentence production within a discourse context compared to stimulation of Wernicke's or sham stimulation (17, 22).

Despite the promising findings of these studies, the evidence-base for tDCS and discourse recovery remains limited. Presently there is a wide range of existing interventions for improving discourse production in chronic aphasia, which implement a variety of treatment methods (5). Some may have a word-level focus and aim to improve lexical retrieval, while others aim to improve aspects of sentence production such as syntactic structure within the discourse context (5). The effect of combining anodal tDCS with different discourse treatments is needed to better understand how best tDCS may be used to support discourse recovery, as currently studies have primarily been limited to the combination of tDCS with conversational therapy.

Therefore, the aim of the present study was to determine the feasibility of anodal tDCS as an adjunct to the Verb Network Strengthening Treatment (VNeST) to improve discourse production in people with post-stroke chronic aphasia. VNeST is a SLT treatment known to improve verb retrieval in discourse production in aphasia (23, 24). As the VNeST in combination with tDCS has not been previously examined, feasibility research is required before proceeding to a definitive randomized controlled trial to examine whether (a) it is feasible to combine VNeST with tDCS; and (b) the intervention shows potential in post-stroke chronic aphasia (25–27). Feasibility was assessed with screening, eligibility, retention, and completion rates, and adverse events (27, 28). Preliminary evaluation of response to intervention was also explored (27, 29) by investigating the impact of VNeST with and without tDCS on discourse production and functional communication, quality of life, psychological state, and cognitive function.

## Methods

### Design

This was a double-blinded, randomized feasibility study. The study protocol was approved by the King's College London Research Ethics Review Board. Written informed consent was obtained from all participants and study procedures were in accordance with the Declaration of Helsinki (30).

Participants were randomly allocated using an online randomization service (31) into an experimental group that received a 6-week block of once weekly, SLT sessions combined with active anodal tDCS or a control group that received the same 6-week block of SLT but with sham tDCS. Assessments were carried out pre-, immediately post-treatment and after 6 months. Sessions took place at the participant's home or at the research lab at King's College London.

### Participants

Six community-dwelling participants with post-stroke chronic aphasia were recruited from community stroke support and communication groups, and community posters between November 2019 and February 2020. Participant inclusion criteria were: (a) mild-moderate aphasia caused by a single stroke, identified by using the Language Screening Test (32, 33) (scores of 0–5/15 = severe, 6–10/15 = moderate, 11–14/15 = mild); (b) ≥ 6 months post onset (c) ≥18 years old; (d) English as a primary language; (e) right handed prior to stroke; (f) normal aided or unaided visual acuity; (g) and willing to participate and comply with the proposed block of intervention and testing regime. Exclusion criteria were persons with (a) neurological symptoms or history of a neurological event other than stroke; (b) history of more than one stroke (c) contraindications to tDCS (i.e., history of seizures, pacemakers); (d) global/severe aphasia, as the language intervention in the study required a higher level of comprehension than is usually observed in people with severe or global aphasia (34) (e) cognitive impairment as identified by a score <23/30 for the Montreal Cognitive Assessment (35); (f) left-handed dominance prior to stroke; (g) visual problems which interfere with persons' ability to access visual materials (i.e., pictures); (h) inability to attend sessions; (i) English as a second language; (j) and persons who were currently receiving SLT or taking part in a similar study. A pre-tDCS screening questionnaire was completed by all potential participants to confirm study eligibility. Collected information included past medical history (i.e., history of seizures, brain surgery, cardiac pacemaker etc.) and hand use preference prior to stroke in activities such as writing and eating to determine handedness (15).

### Intervention and Blinding

#### tDCS

Stimulation was delivered through a battery driven constant current stimulator (DC-Stimulator Plus, NeuroConn, Ilmenau, Germany) with two (5 X 7 cm) saline-soaked sponges. Using the 10-10 EEG system as a guide for electrode placement, the anode was placed over the LIFG or FC5 and the cathode was placed over the contralateral supraorbital ridge. A constant current of 2 mA was applied for 20 min starting from the beginning of each session as this intensity level and duration has been found to be effective in modulating lower level language production as well as discourse output in healthy older adults and PWA (15, 17–19). A 30s ramping period was applied at the beginning and end of the stimulation period. For participants in the sham group, electrode placement was identical however the tDCS was switched off after 30s. The study mode setting was used to allow both the clinician and the participants to be blinded to the tDCS condition. Prior to randomization, a tDCS code (sham vs. active) was randomly assigned to each group by a member of the research team not involved in treatment sessions or outcome assessment. Based on group assignment, the code was then used to activate the stimulator however neither the therapist nor the participants were aware of the condition it was associated with.

#### Language Intervention

Participants in both groups received 45 min of VNeST for aphasia once weekly for 6-weeks. VNeST is a theoretically driven, semantic treatment which aims to improve word retrieval within different language contexts, including discourse, by strengthening semantic connections between verbs (i.e., chop) and relevant thematic roles or “agents” (i.e. subjects such as chef) and “patients” (i.e. objects such as onions) (23, 24, 36). The main treatment step in VNeST is asking participants to produce subject and object nouns to create three different scenarios for each target verb. Producing these scenarios and reading them aloud strengthens the semantic representation of the target verb and its connection to different thematic roles which promotes accurate lexical retrieval (23). Participants then answer where, when and why questions around a single scenario which further strengthens the semantic network of the target verb. As verb production is fundamental to syntax and sentence production (i.e., subject-verb-object), VNeST also strengthens the identification of predicate argument structure and overall syntax (23, 37). The followed treatment protocol was based on the published Tutorial for VNeST by Edmonds (34) (please refer to the tutorial for a detailed description of the treatment protocol) and was delivered via an iPad (Apple, 2013)^1^ using the Advanced Naming Therapy application (38).

### Outcome Measures

#### Outcomes to Assess Feasibility

- Screening rate (number of participants screened/number of referred participants).
- Eligibility rate (number of participants screened/ number of participants who met the inclusion criteria).
- Retention rate, (number of participants who remained in the study or did not drop out).
- Completion rate of intervention (number of participants who completed all outcome measures/total number of participants).
- Completion rate of follow-up (number of participants who completed all follow-up outcome measures/total number of participants) (28, 39).
- Adverse events.

#### Outcomes to Assess Preliminary Response to Intervention

##### Language Tasks

The following discourse production tasks were completed in each assessment session; pre- and post-treatment and at 6-months follow-up.

- Picture description of a street scene – participants were provided with a composite picture depicting a busy street and asked to describe what they see (40).
- Description of a simple procedure such as making a cup of tea (15, 41).
- Cinderella story-participants were asked to tell the story of Cinderella from their memory (15, 41, 42).

Language produced from each discourse task was audio recorded and orthographically transcribed for analysis. The Quantitative Production Analysis procedure was used to extract the discourse samples which were then analyzed for language quantity and complexity (15, 43, 44).

##### Verb Retrieval Outcome Measures

Since Discourse treatment is a newly emerging field in aphasia rehabilitation, there currently is a lack of information on the psychometric properties for the majority of existing discourse production measures and no consensus on which measures are optimal for discourse treatment (5, 45, 46). Therefore, researchers and clinicians are advised to select outcome measures which are aligned with the focus of the implemented language intervent ion (47). The aim of VNeST is to improve verb production, thus the main language measure of interest was verb retrieval in discourse production. This outcome was evaluated using three different measures which have been used in previous research using similar language samples and have been reported to have a high level of inter-rater reliability (17, 42, 48). These included verb token total or the total number of all verb occurrences in a language sample and verb type total or the total number of distinct verbs in a sample (only the first occurrence of each verb produced is counted). The verb type token ratio (VTTR) was then calculated by dividing the verb type total (total number of unique verbs) by the verb token total (total of all verb occurrences) (42). The VTTR is a measure of lexical richness, where a ratio closer to one indicates greater diversity in verb production (49).

##### Secondary Outcome Measures

VNeST is expected to improve word retrieval and overall syntax, thus discourse measures of language quantity also included total number of words and total number of utterances or sentences (44, 50), whereas syntactic complexity was measured using Predicate Argument Structure (PAS) (45). The word count only included words in the discourse sample that were phonologically accurate and directly related to the discourse task. To determine the number of utterances within a sample, prosodic patterns from audio recordings such as evident pauses (i.e., 5 s) and change in intonation or pitch and stress (i.e., falling intonation to mark the end of an utterance) were used to establish utterance boundaries and determine the total number of utterances produced (43, 44, 50, 51). As predicate argument structure is an important element of VNeST, syntactic complexity was measured using PAS, a discourse measure recently identified as one of only four discourse measures with known robust psychometric properties (acceptability, reliability, and validity (45). The PAS is calculated by dividing the total number of arguments by the number of main verbs in a language sample (45).

Aphasia is known to have a significant negative effect on functional communication, quality of life and psychological well-being (52–54), and in the recently published Research Outcome Measurement in Aphasia (ROMA) consensus statement (55), these factors were recognized as key outcomes in aphasia research. Therefore, the following participant reported outcome measures assessing functional communication, quality of life, and psychological symptoms were also completed:

1) The Communicative Effectiveness Index (CETI), is a measure of change in functional communication ability in adults with aphasia that has been reported to have high internal consistency, moderately high test-retest reliability and good concurrent validity (56). This assessment includes 16 everyday situations (i.e., having a one-to-one conversation, giving yes or no answers appropriately etc.). Participants were asked to rate their ability in each communicative situation using a rating scale from 1–10 with one end labeled as “not at all able” and the other “as able as before” (9, 57). Based on previous research a change in CETI scores which indicates a clinically meaningful change or the minimal amount of change that clinicians view as demonstrating improvement in CETI scores (58) has been reported as an increase in at least 12 points (57, 59).
2) The Aphasia Impact Questionnaire-21 (AIQ), (60) is a self-report quality of life questionnaire that utilizes pictures to enable people with aphasia to communicate their experiences of aphasia. The AIQ is one of a few validated assessments to describe both language ability and life with aphasia from the perspective of stroke survivors. The questionnaire has three sections: communication, participation, and well-being/emotional state. The first section looks at activities which are commonly difficult for people with aphasia such as talking and understanding. The participation section focuses on how communication difficulties arising from aphasia impact the person's ability to complete tasks in everyday life such as shopping, and work and the last section looks at the emotional impact of aphasia. Pictural responses in each section convert to a numerical score, where a higher score indicates a greater impact of aphasia on everyday life.
3) Hospital Anxiety and Depression Scale (HADS) is a 14-item scale which assesses non-somatic anxiety (HAD-A) and depression (HAD-D) symptoms that has been reported to have good sensitivity and specificity and has been administered to stroke survivors with aphasia (61, 62). Scores range from 0–21 for each subscale with a score ≥8 proposed for the identification of caseness, for both depression and anxiety in patients with various health conditions (63).

As language function is closely related to other cognitive functions and post-stroke aphasia is usually accompanied by non-linguistic cognitive difficulties such as reduced attention and memory (64, 65) changes in cognitive skills were also examined. Participants completed The Montreal Cognitive Assessment (MOCA) at baseline and post-treatment. The MOCA is an accurate and reliable cognitive screen that has been utilized as a cognitive outcome measure in previous studies to assesses multiple aspects of cognition (i.e., memory, executive function, visual-spatial ability, and orientation) and can be applied in mild-moderate post-stroke aphasia (66).

### Statistical Analysis

Data analysis was performed using SPSS 27 (IBM Inc.) and Prism 9 (GraphPad Software, Inc.). Descriptive statistics were used, and data was assessed for normality. Due to the small sample size and low statistical power the use of inferential statistics was not appropriate. Exploratory analyses were applied in order to examine the potential impact of VNeST combined with tDCS, and whether the intervention shows promise of being successful in post-stroke chronic aphasia (27, 29). Cohen's d effect sizes were calculated to determine an estimate of the likely magnitude of change which provides evidence that the intervention is working as planned (67). Reliable change index (RCI) calculations (27, 68, 69) were used to compare within-group and individual differences. An RCI of 1.96 or greater indicates a significant or reliable change in individual scores.

## Results

### Participants

**Figure 1:**
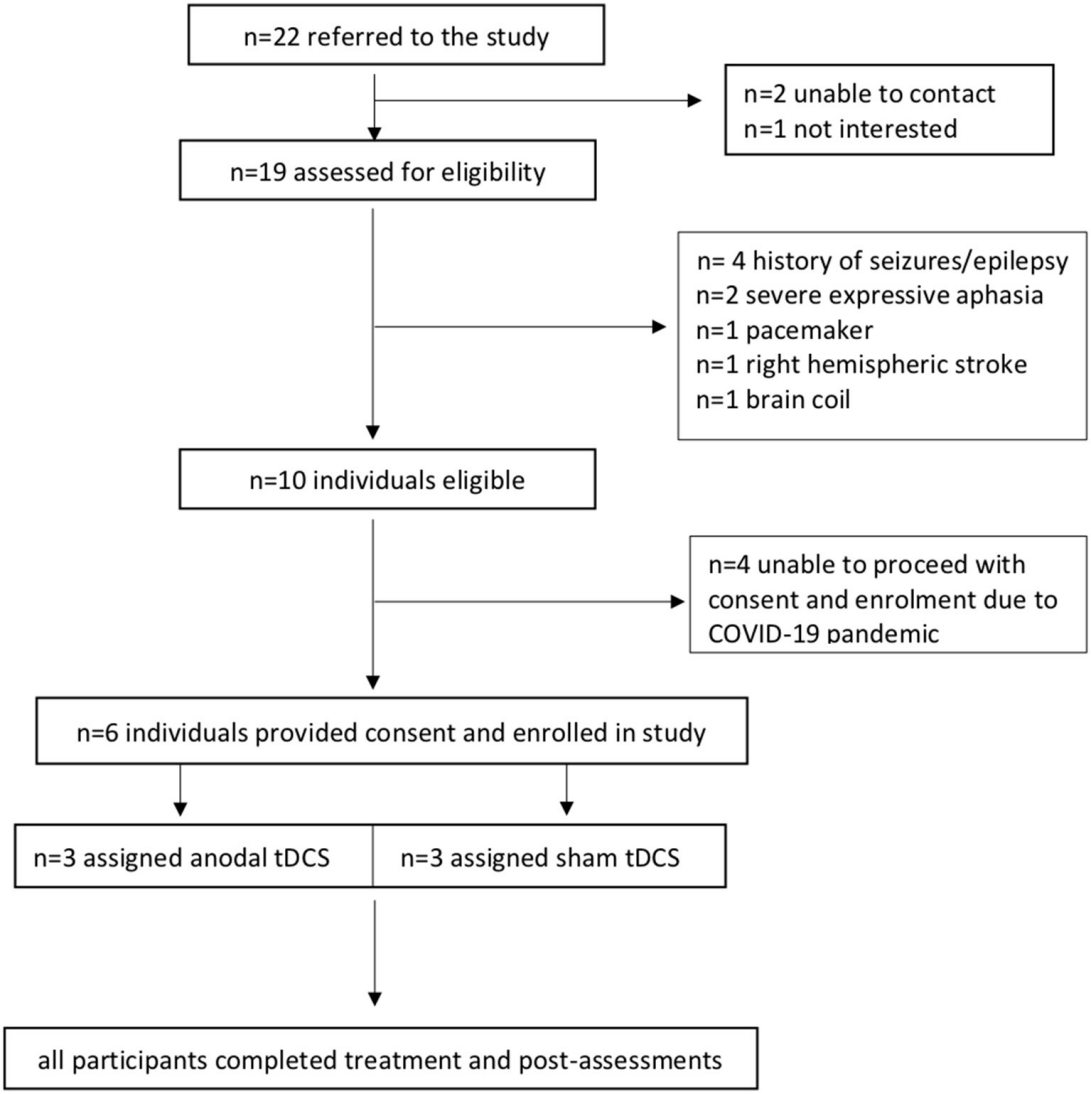
CONSORT flowchart of participants.

Figure 1 presents the study CONSORT flowchart for participant inclusion. Six participants (two males) with post-stroke chronic aphasia were included in this study. The mean age was 71.7 (min = 60, max = 83, SD = 7.3). The main reason for exclusion was tDCS contraindications (Figure 1). Participant demographics are presented in Table 1. Due to difficulty with sourcing detailed neurological data from all community participants, localization of stroke lesions could not be reported (70).

**Table 1:** Demographic and clinical details of participants in both groups.

**Participants**	**tDCS** **group**	**Sex**	**Age range**	**Education level**	**Time post-onset**	**Type of aphasia**	**Severity of aphasia**	**HADS-A (baseline)**	**HADS-D (baseline)**	**MOCA (baseline)**
P1	A	M	70–74	School	1 y, 4 m	Broca's	Moderate	2	1	24
P2	A	F	80–84	University	1 y, 8 m	Broca's	Moderate	5	9	24
P3	A	F	70–74	University	2 y	Anomic	Mild	2	0	23
P4	S	M	70–74	School	1 y, 5 m	Anomic	Mild	4	3	23
P5	S	F	60–64	University	1 y, 4 m	Anomic	Mild	12	10	27
P6	S	F	70–74	School	1 y, 1 m	Broca's	Moderate	3	2	24

*A, active tDCS; S, sham; M, male; F, female; HADS-A, Hospital Anxiety and Depression Scale-anxiety subscale; HADS-D, Hospital Anxiety and Depression Scale-Depression subscale; MOCA, The Montreal Cognitive Assessment. Severity of aphasia identified using Language Screening Test scores (32, 33), scores of 0–5/15 = severe, 6–10/15 = moderate, 11–14/15 = mild*.

### Feasibility Results

The study screening rate was 86%. Out of the 22 individuals referred to the study, 19 were assessed for eligibility (unable to be contacted *n* = 2, not interested *n* = 1). The eligibility rate was 53%. Nineteen individuals were screened and 10 met the inclusion criteria. From the 10 that met the inclusion criteria, six individuals provided consent and took part in the study, giving a consent rate of 60%. Due to the COVID-19 pandemic the researchers were unable to proceed with the other four eligible potential participants who wished to proceed. Participant retention and completion rates for all outcome measures including follow-up testing were 100%. No adverse effects were observed or reported during the treatment. All participants tolerated tDCS.

### Response to Intervention-Between-Group Results

Estimates of magnitude of change using Cohen's d calculations (presented below) revealed large effect sizes in discourse production measures across the three language tasks, with greater change scores noted in the active tDCS group which are summarized below. Between-group results are presented in Table 2.

**Table 2:** Effect sizes comparing pre- to post-treatment and pre-treatment to follow-up change in mean scores between-groups on outcome measures.

	**Change in mean scores pre-post**			**Change in mean scores pre- to follow-up**		
	**Active tDCS** ***n* = 3**	**Sham** ***n* = 3**	**Effect size**	**Active tDCS** ***n* = 3**	**Sham** ***n* = 3**	**Effect size**
**Measure**	**M (SD)**	**M (SD)**	**Cohen's d**	**M (SD)**	**M (SD)**	**Cohen's d**
**Picture description**						
Total words	−12.67 (42.10)	−35.00 (71.90)	0.38	−13.00 (47.15)	−46.00 (79.68)	0.50
Total utterances	1.67 (1.53)	−4.33 (8.08)	**1.03**	1.00 (0)	−6.33 (9.29)	**1.12**
Verb token total	4.33 (4.16)	−11.67 (24.54)	**0.91**	0 (3.00)	−5.33 (10.69)	0.68
Verb type total	7.00 (6.00)	−1.33 (6.66)	**1.31**	4.67 (2.89)	−4.33 (6.81)	**1.72**
VTTR	0.08 (0.08)	0.20 (0.24)	0.67	0.07 (0.06)	−0.08 (0.04)	**2.94**
PAS	0.01 (0.11)	0.08 (0.18)	0.47	−0.02 (0.30)	0.09 (0.21)	−0.43
**Procedural**						
Total words	−2.33 (32.15)	−1.33 (15.95)	−0.04	−20.33 (70.74)	−8.00 (9.54)	−0.24
Total utterances	−1.33 (10.69)	−0.67 (4.04)	−0.08	−1.00 (12.77)	0 (3.61)	−0.11
Verb token total	9.00 (5.29)	−0.67 (6.43)	**1.64**	0.67 (4.93)	−2.63 (4.73)	0.68
Verb type total	3.00 (3.00)	−0.67 (3.79)	**1.07**	1.00 (1.73)	−1.67 (2.89)	**1.12**
VTTR	−0.23 (0.29)	−0.01 (0.10)	–**1.01**	0.03 (0.18)	0.04 (0.07)	0.07
PAS	−0.01 (0.40)	−0.10 (0.35)	0.24	0.06 (0.26)	−0.08 (0.46)	0.38
**Cinderella narrative**						
Total words	43.00 (52.74)	−14.67 (44.56)	**1.18**	77.67 (115.54)	−19.67 (46.29)	**1.11**
Total utterances	7.00 (6.24)	−1.33 (4.16)	**1.57**	11.67 (12.42)	−1.67 (3.51)	**1.46**
Verb token total	17.67 (7.09)	−9.33 (17.93)	**1.98**	18.00 (23.52)	−8.67 (17.79)	**1.28**
Verb type total	11.00 (3.61)	−2.67 (8.96)	**2.00**	13.00 (14.93)	−3.00 (8.54)	**1.32**
VTTR	−0.13 (0.18)	0.11 (0.06)	–**1.79**	−0.20 (0.33)	0.11 (0.23)	–**1.09**
PAS	0.02 (0.24)	0.01 (0.08)	0.06	−0.16 (0.50)	−0.11 (0.08)	−0.14
**Questionnaires/screens**						
CETI	7.33 (2.89)	3.33 (3.51)	**1.24**	11.33 (2.65)	0.33 (1.15)	**5.39**
AIQ	0 (5.00)	−2.00 (1.00)	0.56	NA	NA	
HADS-A	−0.67 (1.15)	1.00 (1.73)	–**1.14**	NA	NA	
HADS-D	1.00 (1.73)	1.00 (4.36)	0	NA	NA	
MOCA	0.33 (0.58)	0.33 (0.58)	0	NA	NA	

*Effect size: small effect = 0.2, medium effect = 0.5, large effect = 0.8; a decrease in AIQ score is a positive gain; Bold items indicate large effect; NA, Not Applicable*.

#### Verb Retrieval Measures

A large effect was found from pre- to post-treatment for the picture description (verb token total d = 0.91; verb type total d = 1.31), procedural (verb token total d = 1.64; verb type total d = 1.07), and narrative (verb token total d = 1.98; verb type total d = 2.00) tasks with only the active tDCS group showing improvement in the number of verbs produced for all three discourse production tasks. At 6-months follow-up a large effect for the active tDCS group was noted in picture description (verb type total d = 1.72; VTTR d = 2.94), procedural (verb type total d = 1.12), and narrative tasks (verb token total d = 1.28; verb type total d = 1.32). A large effect with better results for the sham group in VTTR was found in the procedural task from pre-to post-treatment (d = 1.01) and narrative task from pre-to post-treatment (d = 1.79) and pre-treatment to 6-month follow-up (d = 1.09).

#### Total Words & Utterances

For total number of words, Cohen's d indicated a large effect from pre- to post-treatment (d = 1.18) and from pre-treatment to 6-month follow-up (d = 1.11) with greater improvement for the active tDCS group in the narrative task. For total utterances, a large effect was found from pre- to post-treatment (d = 1.03) and pre-treatment to 6-month follow-up (d = 1.12) for the picture description task with better results for the active tDCS group. Gains were most marked in the narrative task, where Cohen's d showed a large effect from pre- to post-treatment (d = 1.57) and pre-treatment to 6-month follow-up (d = 1.46) with greater improvement in the active tDCS group.

#### PAS

Cohen's d calculations indicated a trivial difference between the groups for this measure of language complexity in all three discourse production tasks.

#### CETI

A more marked improvement in CETI scores from pre- to post-treatment in the active tDCS group compared to sham was evident (d = 1.24). The largest improvement in scores was from pre-treatment to 6-month follow-up in the active tDCS group (d = 5.39).

#### AIQ, MOCA, & HADS

No significant between-group differences were found in AIQ or MOCA scores. For HADS-A scores, Cohen's d calculations revealed a large effect (d = 1.14) from pre- to post-treatment with better results for the active tDCS group. No between-group difference was noted for the HADS-D score.

### Response to Intervention-Within-Group Results

**Figure 2:**
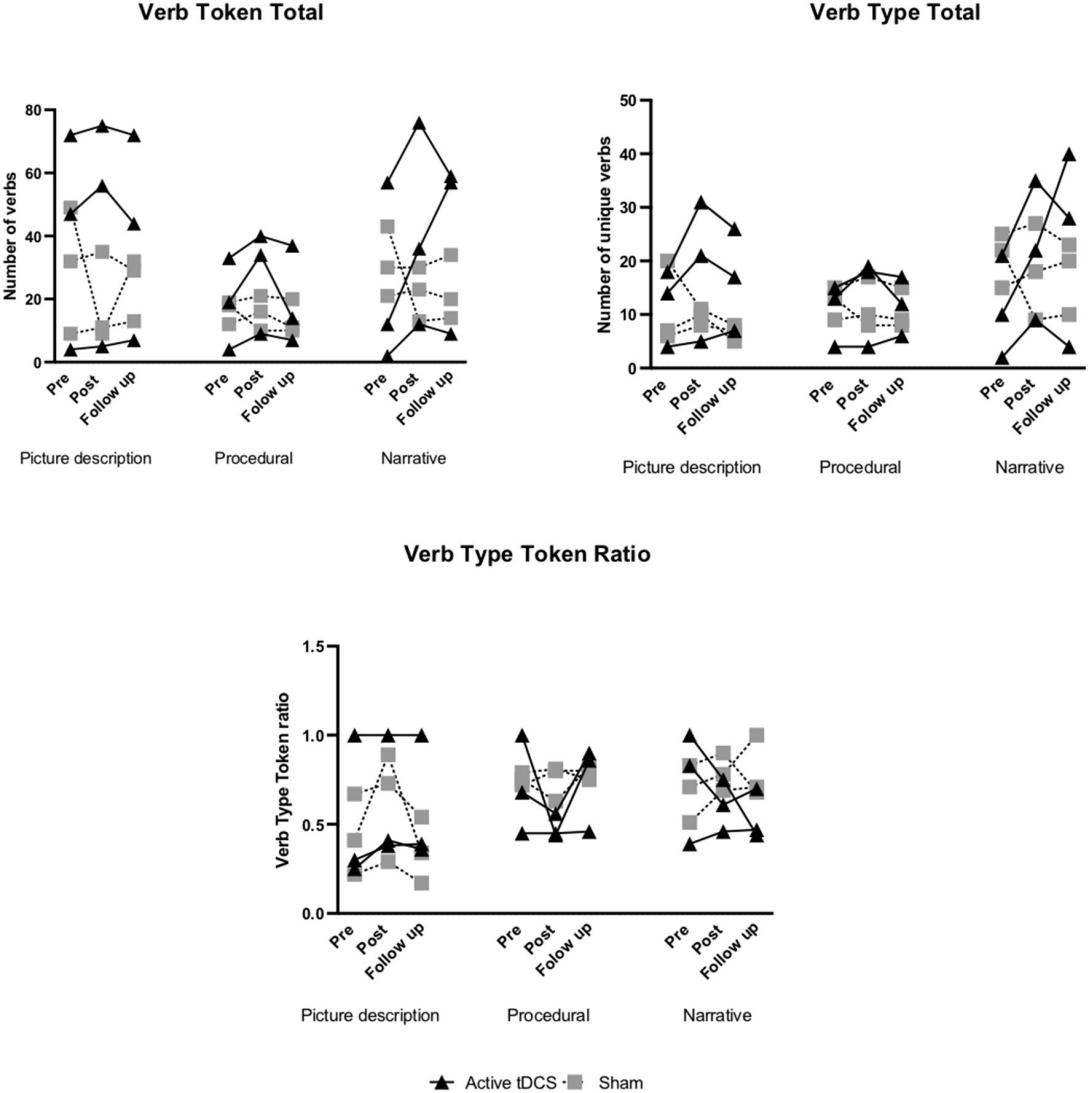
Pre- to post-treatment and pre-treatment to follow-up individual changes in verb retrieval measures: verb token total, verb type total, and verb type token ratio.

**Figure 3:**
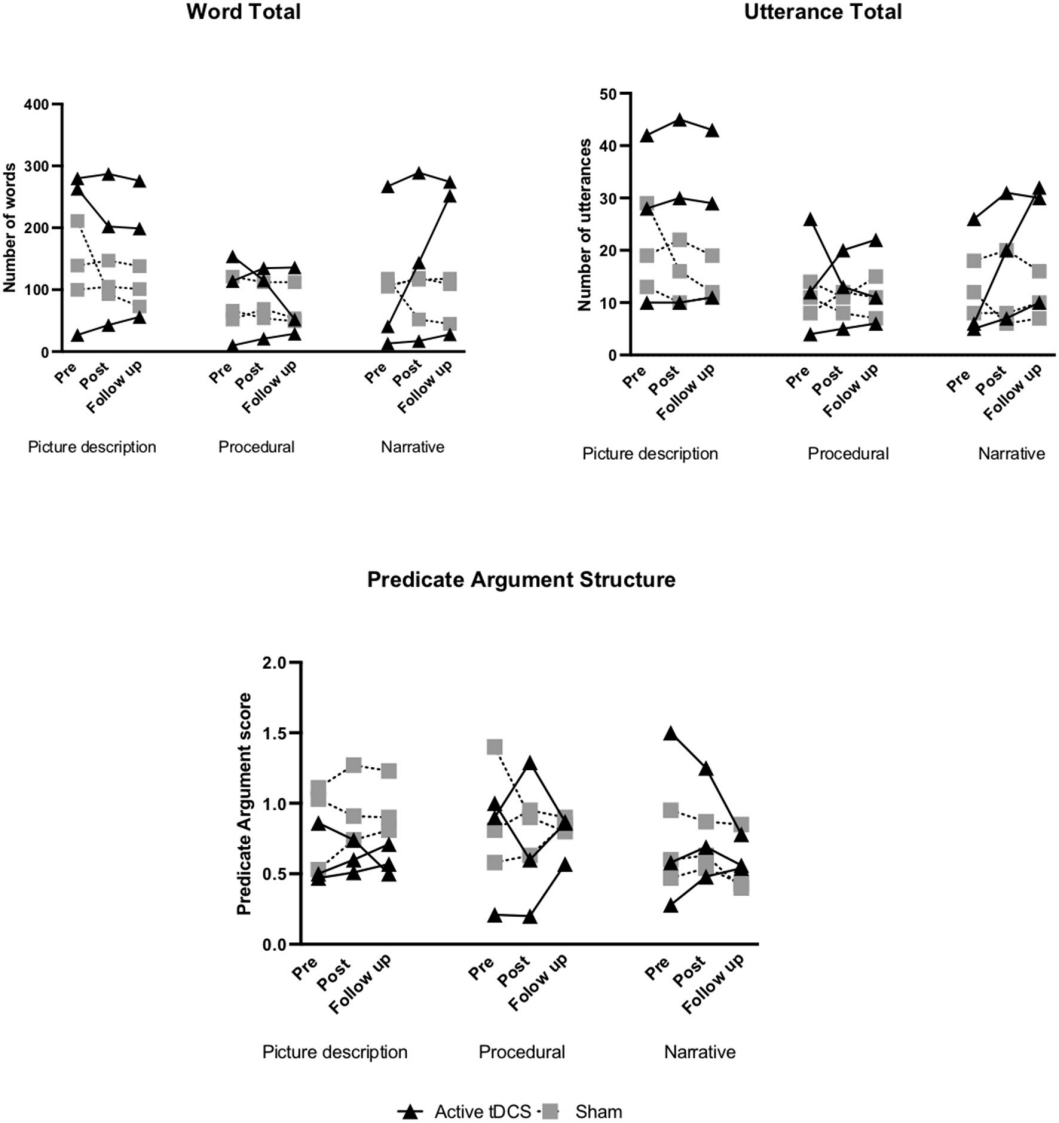
Pre- to post-treatment and pre-treatment to follow-up individual changes in secondary discourse measures: word total, utterance total, and predicate argument structure.

**Figure 4:**
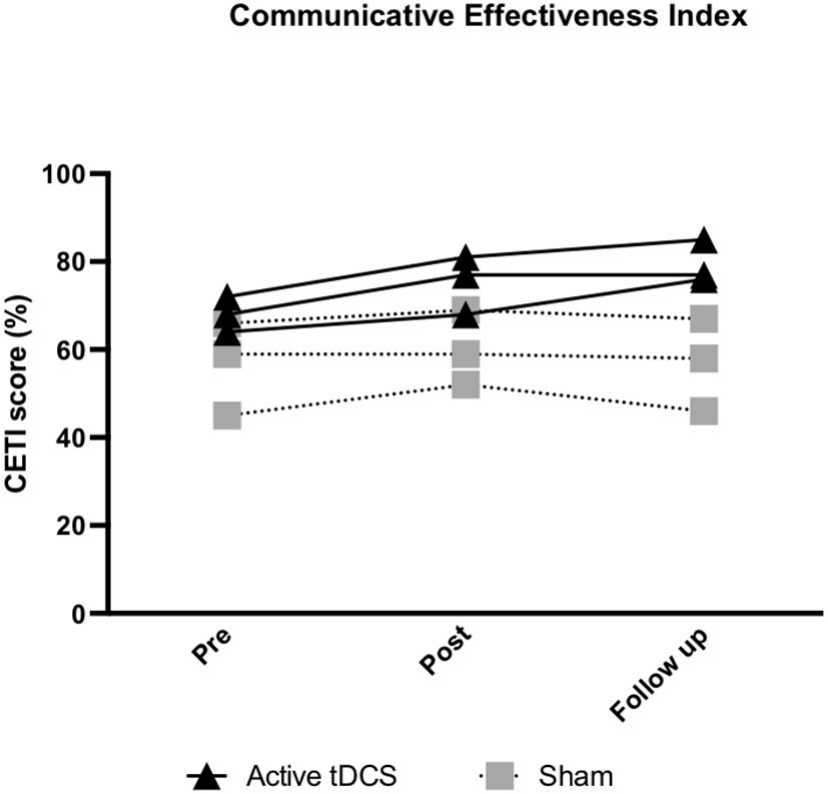
Pre- to post-treatment and pre-treatment to follow-up individual changes in functional communication (communicative effectiveness index; CETI). A higher CETI score indicates improvement in functional communication.

**Figure 5:**
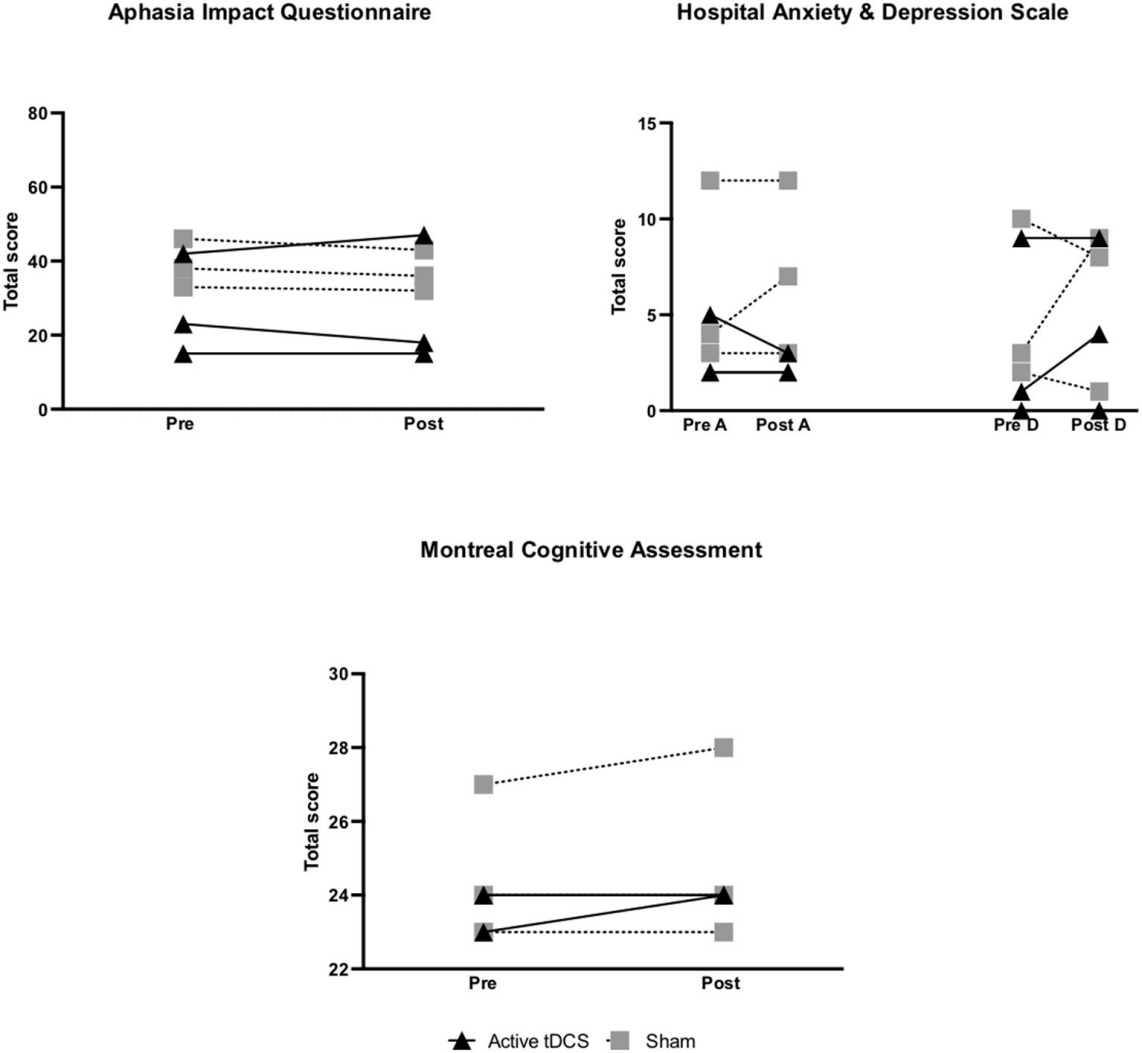
Pre- to post-treatment individual changes in secondary outcomes: quality of life (Aphasia Impact Questionnaire; AIQ), psychological symptoms (Hospital Anxiety and Depression Scale; HADS), and cognition (Montreal Cognitive Assessment; MOCA). A higher score on the AIQ indicates a greater impact of aphasia on quality of life and on the HADS indicates more symptoms of anxiety/depression. For the MOCA, a higher score indicates improved cognitive ability.

Within-group changes in both groups from pre- to post-treatment and pre-treatment to follow-up are presented in Figures 2–**5** and Supplementary Tables 1, 2. Individual participant improvements based on RCI calculations for each group for each outcome measure across the three discourse production tasks are summarized below (Figures 2–**5**). Detailed individual findings are presented in Supplementary Tables 1, 2.

#### Verb Token Total

In the tDCS group 3/3 participants showed a reliable positive change from pre- to post-treatment in all three discourse tasks (picture description, procedural, narrative). At 6-month follow-up 1/3 improved in the picture description and narrative task, while for procedural 2/3 showed a reliable positive change (Supplementary Table 1; Figure 2). No significant improvements were noted in the sham group across the three discourse tasks (Supplementary Table 2; Figure 2).

#### Verb Type Total

In the tDCS group 3/3 participants showed improvement from pre- to post-treatment and 1/3 at 6-month follow-up for the narrative task. For picture description and procedural, 2/3 showed improvement from pre- to post-treatment. At 6-month follow-up 3/3 participants in the picture description task, and 2/3 participants in the procedural task showed positive change (Supplementary Table 1; Figure 2). In the sham group, no reliable improvements were noted for all discourse tasks (Supplementary Table 2; Figure 2).

#### VTTR

In the active tDCS group for picture description, 2/3 participants showed significant improvement from pre- to post-treatment and at 6-month follow-up. For the procedural and narrative task no reliable improvement was noted from pre- to post-treatment. At 6-month follow-up, 1/3 participants showed a positive change in the procedural task (Supplementary Table 1; Figure 2).

In the sham group 3/3 participants showed a reliable improvement in the narrative task from pre- to post-treatment with no positive changes noted at 6-month follow-up. For the picture description task 1/3 participants showed positive change from pre- to post-treatment, however this was not maintained at 6-month follow-up. No significant improvements were noted for the procedural task (Supplementary Table 2; Figure 2).

#### Total Words

In the active group 1/3 participants showed improvement from pre- to post-treatment and at 6-month follow-up for the narrative task. For picture description 1/3 showed positive change at 6-month follow-up. No reliable positive change was noted for the procedural task (Supplementary Table 1; Figure 3).

In the sham group no reliable improvements were noted for the picture description and narrative task from pre- to post-treatment and at 6-month follow-up. For the procedural task 1/3 showed positive change from pre- to post-treatment with no reliable improvements at 6-month follow-up (Supplementary Table 2; Figure 3).

#### Total Utterances

In the active tDCS group 2/3 participants showed positive change from pre-to post-treatment in the picture description and narrative task. At 6-month follow-up 1/3 showed improvement in the narrative task. For the procedural task, no reliable improvements were noted (Supplementary Table 1; Figure 3). In the sham group, no reliable improvements were noted for all discourse tasks (Supplementary Table 2; Figure 3).

#### PAS

In the active tDCS group 1/3 participants showed positive change from pre- to post-treatment in the narrative task. For procedural 1/3 showed a reliable improvement at 6-month follow-up. No significant improvements were noted for the picture description task (Supplementary Table 1; Figure 3).

In the sham group for picture description 2/3 showed positive change from pre- to post-treatment and 1/3 improved at 6-month follow-up. For the narrative task 1/3 showed improvement from pre- to post-treatment. No significant improvements were found for the procedural task (Supplementary Table 2; Figure 3).

#### CETI

In the active tDCS group 3/3 participants showed a reliable positive change from pre- to post-treatment. Furthermore, 2/3 in the active group showed a clinically meaningful change at 6-month follow-up, with participant (P) 1's score increasing by 12 points and P2's by 13 points. Although not clinically meaningful, P3 in the active tDCS group also showed a reliable improvement in CETI scores at 6-month follow-up (RCI = 10.06) (Supplementary Table 1; Figure 4).

In the sham group 2/3 participants showed improvement from pre- to post-treatment and at 6-month follow-up (Supplementary Table 2; Figure 4).

#### AIQ

In the active group 1/3 showed a reliable improvement from pre- to post-treatment (Supplementary Table 1; Figure 5). In the sham group 3/3 participants showed positive change from pre- to post-treatment (Supplementary Table 2; Figure 5).

#### HADS

Both group baseline scores were normal. In the active tDCS group for HADS-A, 1/3 participants showed a reliable reduction in anxiety symptoms. No reliable improvements were noted for HADS-D (Supplementary Table 1; Figure 5).

No improvements were noted in HADS-A and HADS-D scores in the sham group (Supplementary Table 2; Figure 5).

#### MOCA

No reliable positive change was noted in the active tDCS group. In the sham group, 1/3 participants showed an improved score post-treatment (Supplementary Tables 1, 2; Figure 5).

## Discussion

This double-blinded, randomized feasibility study aimed to examine the feasibility, including the preliminary participant response to intervention, of a combined anodal tDCS and SLT intervention on discourse production skills in post-stroke chronic aphasia. There were no adverse reactions, and all participants tolerated the use of tDCS as an adjunct to language therapy. To the best of our knowledge this is the first study to investigate the feasibility of VNeST combined with anodal tDCS on different types of discourse language, functional communication skills, quality of life, psychological symptoms, and cognition. Overall, study findings support the feasibility of using tDCS alongside VNeST. Exploratory analyses revealed promising changes (i.e., estimated large effect size) in discourse production measures across discourse language tasks and functional communication for the active tDCS group.

Recruitment to stroke trials has been reported as challenging and everyday issues (i.e., commuting to study sessions) can hinder the process of recruiting people with aphasia (71). Despite these challenges, study retention and completion rates for all outcome measures including at 6-month follow-up were 100% which supports the feasibility of tDCS combined with VNeST as an intervention. The screening rate was high (86%) and greater than 50% of screened individuals met inclusion criteria, indicating the study was representative of the target population (39). However, many screened individuals (47%) were not suitable mainly due to tDCS contraindications. These findings indicate that tDCS contraindications may be common in stroke survivors, thus this should be taken into account when planning future studies as recruitment may be hindered. Overall, feasibility data provides impetus for a future large-scale study.

This feasibility study did not have sufficient power to test the effectiveness of tDCS combined with VNeST. Nevertheless, our preliminary findings suggest that it is feasible to apply anodal tDCS over the LIFG as an adjunct to VNeST and tDCS may have the potential to boost VNeST treatment outcomes. These findings provide further support for the exploration of tDCS in post-stroke chronic aphasia (72). Our early findings demonstrate that anodal tDCS combined with VNeST may possibly result in improvements in verb retrieval, and other measures of language quantity (total number of words, total number of utterances) at discourse level. Both groups showed improvement in the richness of verbs produced (VTTR) as expected as the VNeST intervention is known to improve verb retrieval at discourse level for trained and untrained items (24). However, only the active tDCS group demonstrated a significant improvement (large effect size) in verb retrieval (verb token total, verb type total) across all three discourse production tasks. This novel finding supports the potential of this combined tDCS treatment in improving verb production, a major difficulty for many with aphasia (42), across discourse genres.

Our results further support previous findings of combined SLT and tDCS studies for improving language quantity at the discourse level. Marangolo et al. combined conversational therapy with anodal tDCS over the LIFG which resulted in a significant improvement in the number of verbs and sentences produced during videoclip descriptions compared to sham condition (22). Similarly, Campana et al. combined anodal tDCS over LIFG with conversational therapy which resulted in a significant improvement in verb naming during picture description (18). Although improvements in language quantity were evident in the active tDCS group compared to sham in this study, effect size calculations showed insignificant differences between groups for language complexity, suggesting that anodal tDCS may have a greater impact on language quantity. However, the small number of participants in this study, although similar to previously published studies in aphasia research (7) and tDCS feasibility studies in stroke (73), limits the interpretation of these results. As this was a feasibility study, a formal sample size calculation was not appropriate. A future pilot study with at least 12 participants per group (74, 75) would allow for a formal sample size calculation for a full-scale study and clearer understanding of the effect of tDCS on language quantity and complexity in discourse production.

Previously the exploration of tDCS effects on everyday communication has been very limited (72), however our preliminary results indicate that anodal tDCS may improve functional communication in post-stroke chronic aphasia. Unlike a recent review which found no evidence of tDCS improving everyday communication (76), our exploratory analyses revealed a significant difference post-treatment and at 6-month follow-up between- and within-groups in CETI scores, indicating a potential for anodal tDCS application to result in improvements in functional communication. Furthermore, two of the three participants in the active tDCS group showed clinically meaningful change in functional communication at 6-month follow-up. These findings are in agreement with a previous RCT assessing the effect of a combined intensive naming therapy with anodal tDCS which also resulted in significant improvements in everyday communication as measured by the CETI (9). Contrarily, Guillouët et al. found that application of bi-hemispheric tDCS (anode on LIFG, cathode on contralesional IFG) did not significantly improve spontaneous speech, an important functional communication skill (7). The assessment of spontaneous speech however only involved assessing change within a single response to an open-ended question, whereas the CETI, a validated functional communication measure, covers a wide range of everyday communication scenarios (57). Although bi-hemispheric stimulation may have a positive effect on naming (7, 77), the difference in our results suggests that it may not have the same impact on higher level language skills and uni-hemispheric stimulation may be the optimal choice for targeting discourse production.

The lasting effects of tDCS has recently been a topic of interest. The aftereffects of single session tDCS are transient, lasting up to an hour, whereas effects of multiple stimulations can last for days and months (9, 78). The findings of this study, the first to examine the long-term effect (6 months) of anodal tDCS on discourse production, provide further support that the impact of multiple tDCS sessions may be long lasting. The majority of large gains in the active tDCS group were maintained at 6-months and for some measures further enhancements were noted at this time. These findings suggest that neuroplastic aftereffects of anodal tDCS may potentially lead to optimum improvement months after stimulation, highlighting the importance of longer follow-up periods. The mechanism underlying the effects of repeated tDCS sessions applied concurrently with behavioral training is believed to be similar to long term potentiation (LTP), a critical process for neuroplasticity (9, 79). Imaging studies with healthy speakers have also found that decreased brain activity was associated with improvements in naming ability during application of anodal tDCS over the LIFG (13, 15, 80, 81), suggesting that tDCS may indirectly modulate behavior by increasing processing efficiency (15, 81, 82). Although these mechanisms may rationalize our findings, further research is required to understand the underlying mechanisms of tDCS which allow it to modulate language in persons with aphasia.

Higher anxiety levels have been linked with deficits in communication (15, 83). The preliminary results for the active tDCS group show a reduction in HADS scores for anxiety in only one out of the three participants. Due to these limited findings and the small sample size no conclusions can be drawn regarding whether SLT in combination with anodal tDCS may potentially contribute to lowering anxiety symptoms in post-stroke aphasia. There were no evident improvements in quality of life or cognitive skills in either intervention group. As VNeST is a language therapy aimed at improving lexical retrieval and not typically expected to impact cognitive skills, the lack of change in the MOCA supports that observed post-treatment improvements may possibly be due to VNeST treatment effects. However, larger full-scale evaluation studies are required to establish the effect of tDCS on psychological symptoms, quality of life and cognition in both the short and long term.

Although early exploratory findings were promising, this study had a number of limitations. Due to difficulties with recruitment which were further exacerbated by the COVID-19 pandemic, the sample size was small. As a feasibility study with a small sample size comparative analysis could only be exploratory and thus no valid conclusions could be made from differences between the groups for all of the outcomes in the study (29). Although the outcome assessor was blinded, the lack of an additional assessor did not allow us to establish inter-rater reliability and increased evaluation bias (7). The study also lacked multiple baseline evaluations which would have controlled for possible intraindividual variability commonly seen in post-stroke aphasia (7, 84).

## Conclusion

This study demonstrates that combing anodal tDCS over the LIFG with VNeST for improving discourse production in post-stroke chronic aphasia is feasible and well-accepted by participants. Preliminary findings indicate a potential for the use of tDCS as a safe and inexpensive tool for boosting aphasia rehabilitation in chronic aphasia. As a feasibility study, our findings provide motivation for future large-scale trials in this field to establish the effect of tDCS on discourse production and its potential to be used routinely as a rehabilitative tool in clinical practice.

## Data Availability Statement

The original contributions presented in the study are included in the article/Supplementary Material, further inquiries can be directed to the corresponding author/s.

## Ethics Statement

The studies involving human participants were reviewed and approved by King's College London Biomedical & Health Sciences, Dentistry, Medicine and Natural & Mathematical Sciences Research Ethics Review Board. The patients/participants provided their written informed consent to participate in this study.

## Author Contributions

SM, CN, IS, and MP: study concept. SM, CN, and MP: development of methodology and manuscript draft revisions and editing. SM: data collection and original draft preparation. SM and MP: data analysis. MP: supervision. All authors read and approved the final manuscript.

## Conflict of Interest

The authors declare that the research was conducted in the absence of any commercial or financial relationships that could be construed as a potential conflict of interest.

## Publisher's Note

All claims expressed in this article are solely those of the authors and do not necessarily represent those of their affiliated organizations, or those of the publisher, the editors and the reviewers. Any product that may be evaluated in this article, or claim that may be made by its manufacturer, is not guaranteed or endorsed by the publisher.

## Footnotes

^1^Apple iPad Air iPadOS 12.5.5.
